# Effects of a Fact Sheet on beliefs about the harmfulness of alternative nicotine delivery systems compared with cigarettes

**DOI:** 10.1186/1477-7517-9-19

**Published:** 2012-06-11

**Authors:** Ron Borland, Lin Li, K Michael Cummings, Richard O’Connor, Kevin Mortimer, Tom Wikmans, Lars Ramstrom, Bill King, Ann McNeill

**Affiliations:** 1 VicHealth Center for Tobacco Control, The Cancer Council Victoria, Carlton, Australia; 2 Hollings Cancer Center, Medical University of South Carolina, Charleston, SC, 29425, USA; 3 Roswell Park Cancer Institute, Buffalo, NY, USA; 4 University of Nottingham, Nottingham, UK; 5 Research Group for Societal and Information Studies, Stockholm, Sweden; 6 Institute for Tobacco Studies, Stockholm, Sweden; 7 UK Centre for Tobacco Control Studies, Nottingham, UK; 8 Nigel Gray Distinguished Fellow in Cancer Prevention, VicHealth Center for Tobacco Control, The Cancer Council Victoria, 1 Rathdowne St, Carlton, VIC, 3053, Australia

## Abstract

**Background:**

This study explored the value of providing information in a Fact Sheet to correct misperceptions about the relative harmfulness of nicotine replacement products (NRT) and smokeless tobacco (ST), when compared to cigarette smoking.

**Methods:**

Four convenience samples from different countries (Australia, UK, Sweden and USA) were surveyed concerning their beliefs about the relative harmfulness of smokeless tobacco and NRT. Study participants were given the Fact Sheet that explained that nicotine, as used by consumers, is not particularly harmful and explained why. They were resurveyed one week later regarding their beliefs about the relative harmfulness of smokeless tobacco and NRT and future intentions to use the products.

**Results:**

In all four samples knowledge increased by similar amounts and beliefs regarding the lower harmfulness of smokeless tobacco increased. However, misconceptions remained common and responses to belief measures were not always consistent. Likelihood of use of ST increased in all four samples after exposure to the Fact Sheet, but interest in NRT use only increased in the US sample.

**Conclusions:**

A Fact Sheet such as this one can help address misconceptions about NRT and smokeless tobacco, at least in the short term. However, as is true of most educational interventions, exposure to a single educational session is not sufficient to overcome misperceptions that smokers have about the relative harmfulness of oral versus combustible forms of nicotine delivery.

## Background

The majority of harm from tobacco use comes from combusted forms; that is, from the dirty delivery system, not the nicotine. Some forms of smokeless tobacco (ST) are less harmful than others [1,2], with some existing smokeless products still very harmful (albeit less harmful than smoking) such as oral tobacco products used in South Asia and Sudan [3]. This is because they contain high levels of toxicants, often produced during the manufacturing, curing and storage process. However, where concerted efforts have been made to minimize the levels of known carcinogens and toxicants (most notably with Swedish snus), the potential harms can be reduced dramatically compared to cigarettes and made similar to that of oral nicotine medications, such as gum or lozenge. Cleaner forms of smokeless tobacco have been estimated to be 90–95% less harmful than cigarettes when used long term [4], and some contend they may be even less harmful [5]. Recent research syntheses suggest that the difference in estimates is largely a function of the attributed risk for heart disease and stroke [6-8], as it now appears that there is little or no excess cancer risk [9,10]. If as seems prudent, one assumes the current epidemiology which suggests an elevated risk of both heart disease and stroke [6-8], then the lower estimate is the most plausible. However, if this risk is less or even negligible for the on-average cleaner products such as Swedish snus [7], then the potential reduction would be more consistent with the smaller estimates of risk.

There is now evidence that ST (in at least some forms and in some contexts) can contribute to smoking cessation and is not a pathway to uptake of cigarettes [11-13]. While some report using ST to quit smoking on a short-term basis, others use it as a longer-term substitute or for an intermediate period of use before quitting all nicotine use [11,13-15]. Opponents of marketing smokeless tobacco as a harm reduction alternative to cigarette use argue that partial substitution could sustain longer term use of combustible tobacco thereby negating any harm reduction benefit. Also, the concept of promoting ST as an alternative to smoking could entice non-tobacco users to start using smoked tobacco. However, as Kozlowski has argued, the primary responsibility of health authorities is to ensure that the public is well informed about the relative harms of alternative nicotine delivery systems [16].

Medicinal quality nicotine replacement products (NRT) are the standard by which the cleanest forms of smokeless tobacco should be compared. Currently most forms of NRT are engineered to minimize consumer appeal as regulators see this as increasing abuse liability. This is achieved by avoiding products that allow the rapid absorption of nicotine, a key factor in their consumer attractiveness [17]. The potential of NRT products for long term use is largely untested, although some people who use NRT to quit smoking continue to use it longer term [18]. The limited available evidence is that use of NRT for up to at least 5 years is safe [19].

Some tobacco control advocates have been arguing for a strategy of encouraging smokers to move to alternative forms of nicotine [20]. This is supported by major reports supporting harm reduction strategies [5,21]. For a product substitution strategy to be effective, it would require that tobacco users understand the relative safety of alternative forms of nicotine, be prepared to try them as alternatives, and for them to find the products sufficiently attractive to persist in their use, and also stop using smoked forms.

There is little doubt that in Sweden, the use of ST (in this case Swedish snus) and, to a lesser extent NRT, have contributed to a reduction in the prevalence of smoked tobacco use among men. In Sweden, even with high levels of long-term ST use, there is a net health benefit from the reduction in smoked tobacco as evidenced by the rapid decline in smoking caused deaths [22]. The National Board of Health and Welfare (Socialstyrelsen) stated in its National Public Health Report for 2005 (after summarizing recent scientific studies): *“Hence these results indicate that the net effects of using snuff as a way of stopping smoking can be positive since smoking is so much more hazardous to health than snuff-taking”*[23]. The apparent substitution of ST for cigarettes in Sweden is remarkable in the context of there having been no explicit promotion of ST as a less harmful alternative to smoking. Research from Sweden [24] shows low levels of public knowledge about the relative harms of smoked and smokeless tobacco products, so the movement to ST in Sweden cannot be thought of as a movement driven primarily by harm reduction motives. In this study, only 29% of daily smokers knew that NRT is a lot less harmful, so there are clearly misconceptions about NRT as well.

Lack of understanding regarding the relative harm of smokeless tobacco and NRT compared to cigarettes is also widespread in other countries [25-27]. In three of the countries studied here (US, UK and Australia [26]) awareness of the reduced risk of ST is lower than for NRT, but both are low and held by a minority, except in the UK where a small majority of smokers accept that NRT is low harm. These beliefs have remained constant over most of the 2000s, except in the UK, where they have increased modestly [26].

In this paper we present the results of a small intervention study intended to explore the effects of providing factual information on the relative harms of ST/NRT and smoked tobacco on smokers’ opinions. The Fact Sheet we produced was designed to provide the kind of assessment of the relative risks that health authorities might be persuaded to authorize. We hypothesized that exposure to the Fact Sheet would increase smokers’ knowledge about the relative harmfulness of ST/NRT compared to cigarettes and that this in turn would increase receptivity to using ST/NRT as possible substitutes for cigarettes in the future. We also hypothesized that these findings would be consistent across countries, despite differences in the use and availability of ST/NRT products.

## Methods

A before-after study design was used to assess changes in smokers’ understanding of relative harms of nicotine products attributable to provision of a Fact Sheet summarizing current (at the time it was written) scientific knowledge on the relative harms of nicotine and smokeless tobacco as compared to smoked tobacco.

The Fact Sheet was three A4 pages long: a one page overview and 2 pages of “Commonly asked questions” with answers provided. It was provided in pdf form to download for those completing the study on the internet. In the USA, the Fact Sheet was handed to participants to read and was augmented by a narrated Powerpoint presentation of the material. The content was essentially the same in each country although references to country were tailored to the country of use, and the Swedish version was translated into Swedish by the Swedish co-authors. (A copy of the version used in Australia is appended. See Additional file 1).

Participants: We recruited convenience samples of smokers from four countries using a variety of recruitment techniques (see Table 1). Table 2 provides a description of the study samples recruited in each country.

**Table 1:** Recruitment methods used in each country

**Country**	**Sample size**	**Recruitment of study sample**
Australia	170	In Australia we initially recruited 391 smokers to a survey on a smoking cessation website (http://www.quit.org.au). After the initial survey, smokers of 10+ cigarettes per day who said they were not planning to quit in the next month were told about the possibility of participating in a study that involved trying some alternatives to cigarettes. A total of 170 completed the second survey. Both surveys were completed on the internet.
Sweden	187	In Sweden a sample of 213 daily smokers (without specific selection criteria) were recruited from an internet panel and of these 187 completed the second survey.
United Kingdom	101	In the UK participants were recruited through advertisements in local newspapers about a study on alternatives to cigarettes. 101 of 144 completed the two surveys. Pencil and paper surveys were used.
United States	59	In the US participants were recruited through advertisements in local newspapers about a study on alternatives to cigarettes. 59 of 67 completed the two surveys. In the US sample, subjects attended the research site on both occasions.

**Table 2:** Characteristics of the samples who completed both Pre and Post Fact Sheet surveys

**Participant characteristics**	**Aust n = 170**	**Sweden n = 187**	**UK n = 101**	**US n = 59**
Age (mean ± SD, years)	38.6 (11.1)	41.6 (11.9)	40 (12.6)	47.2 (9.7)
Sex (% males)	31.8	26.2	40.0	44.1
Education (% tertiary education)	38.2	29.4	19.8	20.3
Dependence (% heavy smokers#)	20	3.7	10.8	20.3
Ever use of NRT, % of total	70.0	64.2	60.4	49.2
Ever use of ST, % of total	7.7	40.1	11.0	10.2

NB: # heavy smokers means those who smoked more than 20 cigarettes per day and smoked first cigarette within 5 minutes of waking.

### Measures

The initial survey contained questions on demographics and basic information about smoking. Both surveys contained questions on the relative harmfulness of NRT and ST separately against smoked tobacco (A lot less harmful, A bit less, Similarly harmful, More harmful, Don’t Know (recoded with Similar for some analyses)). Questions regarding the likelihood of using NRT on their next quit attempt, and likelihood of trying ST, were asked, each with 5 options from very likely to very unlikely.

Five questions about the mechanisms by which harms of smoking cigarettes come about were asked and six response options (none, a bit, some, a large amount, a very large amount, and virtually all) were provided for each question. These questions are: 1) how much do they think the harm comes from the chemicals that are in the tobacco before it is burned (with those answered ‘a bit’ or ‘some’ were coded as 1, otherwise coded as 0); 2) from the chemicals that are created by burning the tobacco (‘a large amount’ or ‘a very large amount’ were coded as 1, otherwise 0); 3) from taking the smoke into the lungs (‘some’, ‘a large amount’ or ‘a very large amount’ were coded as 1, otherwise 0); 4) do filters remove the harmful ingredients in tobacco smoke (‘a bit’ was coded as 1, otherwise 0); and 5) the harm is due to the nicotine (‘a bit’ was coded as 1, otherwise 0). This gave a knowledge score of 0–5.

They were also asked about intention to quit (No intention, Open to the possibility, Thinking of quitting, but not in the next month, Planning to quit in the next month, and, I have already quit - follow-up only). At the second survey, respondents were also asked how much of the Fact Sheet they read (None, Skimmed it only, Read some, Read all), ease of understanding (Very easy, Fairly easy, Fairly hard, Very hard) and whether they found the information credible (Totally convinced, Largely convinced, Uncertain, Not convinced, I think it was wrong).

### Statistical analyses

Paired t tests were used to test for changes in knowledge and McNemar’s test for changes in the categorical variables. All analyses were conducted using Stata Version 10.1.

### Ethical approval and consent

The Australian study was approved by the Cancer Council Victoria’s Human Research Ethics Committee (reference no. HREC0625), the UK one by Nottingham Research Ethics Committee 2 (reference no. 07/Q2404/53), and the US one by the Research Ethics Board of the Roswell Park Cancer Institute. In Sweden as this was done as an anonymous web survey, it is exempt from Ethics requirements. A full review by a research ethics committee has not been necessary for this type of data collection according to the rules of the Swedish Law on Research Ethics Review (2003:400). In all four countries written informed consent was obtained from the participants for publication of this report and any accompanying images.

## Results

The main findings are summarised in Tables 3 and 4. Most (around 90% or more) read or at least skimmed the Fact Sheet. Nearly all (over 95%) found it very or fairly easy to understand (not in Table), but there was considerable variation in how convinced they were, with most retaining a degree of scepticism (Table 3).

**Table 3:** Reported levels of having read the Fact Sheet and extent of being convinced, by country

	**Australia (n = 170)**	**Sweden (n = 187)**	**UK (n = 101)**	**US (n = 59)**
**Read Fact Sheet (% of total)**				
No	11.2	3.2	1.0	10.2
Yes, skim/some	51.8	34.2	9.9	33.9
Yes, all	37.1	62.6	89.1	55.9
**Extent convinced (% of Read/skimmed only)**				
Totally	9.8	21.9	9.1	25
Largely	29.3	34.4	36.4	50
Uncertain	57.3	39.1	54.6	25
Not at all	3.7	4.7	0	0

**Table 4:** Data comparison between Pre-test and Post-test, by country

**Question**	**Australia (n = 170)**		**Sweden (n = 187)**		**UK (n = 101)**		**US (n = 59)**	
	**Survey 1**	**Survey 2**	**Survey 1**	**Survey 2**	**Survey 1**	**Survey2**	**Survey1**	**Survey 2**
Intention to quit, (% of total)		***^		NS		NS		NS
Not thinking of quitting	31.2	26.6	46.0	39.0	73.1	63.3	72.9	69.5
Thinking of quitting, but not in the next month	16.5	17.3	42.8	44.4	9.9	9.9	22.0	20.3
Planning to quit in the next month	52.4	32.7	11.2	14.4	16.8	18.8	5.1	10.2
Already quit	−	23.5	−	2.1	−	6.9	−	0
% correct perception of harmfulness of NRT vs cigarettes (If answered ‘a lot less harmful’)	42.9	52.8*!	39.6	42.8NS	55.5	65.4NS	22.0	30.5NS
% correct perception of harmfulness of ST vs cigarettes(If answered ‘a lot less harmful’)	7.7	35.8***!	14.4	28.3***	21.8	53.5***	6.8	27.1**
% Likely to use NRT (next try to quit)	56.3	52.5 NS!	70.8	55.4NS	64.4	75.3*	28.8	63.5***
% Likely to try ST (if available)	26.5	47.2***!	15.1	Not asked	50.5	79.2***	28.8	45.8*
Knowledge score (0–5, mean)	2.06	2.47***#	2.4	2.8***	2.2	2.5*	2.1	2.4 NS

Notes: *Significant at *p* < 0.05;**p < 0.01;***p < 0.001;NS = not significant . ^asymptotic symmetry tests; !McNemar tests; # Paired *t* test results for knowledge scores.

As can be seen from Table 4, knowledge of the mechanisms of tobacco-related harms became more accurate, with a similar magnitude of change in all 4 countries, although this was not significant of itself in the USA. Increases in knowledge were mainly among those who read all or at least some of the Fact Sheet as might be expected. Believing that NRT was far less harmful than cigarettes increased overall, and although the change was only significant in Australia, the increase was similar in the smaller UK and USA samples, but was smaller in Sweden. By contrast, believing that ST was less harmful increased significantly in all countries, although in no case to the level of the corresponding belief about NRT. There was no change in the percentage of respondents saying they were likely to use NRT for their next quit attempt in Australia or Sweden, but there were positive effects in both the UK and USA samples. There were significant increases in interest in trying ST in the three countries where subjects were asked about interest in trying ST (this question was not asked in Sweden as we assumed low levels of never trying).

We also looked to see if more accurate beliefs about the relative harmfulness of the products were related to beliefs about the mechanisms of harm. We found a small but significant effect in Australia where the beliefs became more highly inter-correlated (from -0.09 to +0.18), however in the other three countries this effect was not clear, although trends were mainly in the expected direction. In all cases the relationship between the knowledge score and beliefs about product harmfulness were low. Similarly the relationship between beliefs in harmfulness and likelihood of use increased in Australia (0.18 to 0.28 for NRT and 0.14 to 0.25 for ST), although the increases were not significant.

## Discussion

As hypothesized, exposure to the Fact Sheet improved smokers’ knowledge levels about the relative harmfulness of ST/NRT compared to cigarettes and increased interest in using both products as a substitute for their cigarettes. However, the overall effect of exposure to the Fact Sheet was modest, with many smokers remaining unconvinced about the relative harms and the value of switching from cigarettes to ST/NRT.

It is implausible that the improvements in knowledge and beliefs found here could be due to secular trends. The data was collected over a period of weeks or months in three of the countries (it was done in a short interval in Sweden), and there was no possibility of a common extraneous event between the surveys that could plausibly produce the changes found. Further, findings from the International Tobacco Control (ITC)-Four country study (which covers 3 of the 4 countries studied here) show smaller or no changes in some of these beliefs over a period of years [26]. The low level of understanding reflects a failure of public education, and in some cases probably exacerbated by misleading statements from authorities about relative harms [20].

This study has a number of limitations worth noting. First, we relied on relatively small convenience samples of smokers from each of the four countries. In two of the countries, subjects recruited had some expectation of being offered the opportunity to try some nicotine products, and in a third (Australia), part of the sample was told about such an opportunity between surveys. However, the consistency of the results across the four diverse samples strongly suggests that the Fact Sheet improved knowledge wherever it was read. We would also expect that those who chose to participate in the study (especially returning for the second survey) are probably, as a group more interested in using such products than the general population of smokers, so would have been more motivated to read the Fact Sheet than a random sample of smokers. Thus we cannot use this data to estimate likely population-level impact of disseminating the information in a Fact Sheet such as this.

Second, our findings concern a short period between being exposed to the information and the test for changes in outcomes. We do not know how much of the increased knowledge will persist in the absence of reinforcing information. Evidence from education research typically shows a decline in acquired knowledge with time after a single exposure to new information, so it is likely that there would need to be repeated exposures in order for to the information to permanently alter beliefs, intentions and to have any sustained influence on tobacco use behaviours [18,19]. In this study we found that the belief measures about the relative harmfulness of ST/NRT were not strongly correlated with each other, suggesting that beliefs about the harmfulness of different tobacco products may not be strongly held and are thus more susceptible to change than more strongly held beliefs. Thus, even a relatively modest intervention such as repeated exposure to facts about the relative dangers of different nicotine containing products, coupled with an explanation of why this is biologically plausible, might over time prove to be effective in persuading smokers to reduce their consumption of cigarettes, perhaps with substitution of less dangerous nicotine delivery products. It is also notable that the views of Swedish smokers were very similar to those in the other countries, even though the use of smokeless tobacco is widespread in Sweden.

Related to this, while we found consistent effects of the Fact Sheet leading to increased interest in using smokeless tobacco, we did not find a consistent effect for nicotine replacement, which may be due to greater experience with NRT by smokers and greater awareness of NRT products which have be widely promoted as an alternative to cigarette smoking unlike smokeless tobacco products, but note that where there was face to face contact (the US sample) interest in NRT use increased, but when all contact was via the internet (and thus impersonal) we did not see such an effect.

Third, this study essentially relied upon a text only pamphlet to communicate information to study subjects. This mode of communication was selected since it mimicked what might be attached to cigarette packs. However, using more graphic and persuasive forms of communication which would be repeated in multiple communications channels would surely have produced stronger effects.

Following provision of the Fact Sheet, smokers uninterested in quitting in three of the samples (not Sweden) were offered the possibility of trying some of these products [28,29] and patterns of use and intentions to use again were largely consistent with intentions as expressed after receiving the Fact Sheet.

The results provide evidence that the provision of information in the form of, say, a cigarette pack onset/insert, might be an effective means to educate smokers about the relative harmfulness of alternative nicotine delivery products such as ST and NRT. Given the low levels of knowledge that smokers had about harmfulness of different nicotine delivery products, it would seem to be in the public interest to require such information to be placed on cigarette packs and at the point of sale for tobacco products to ensure that smokers are better informed about the relative harmfulness of smoked and unsmoked nicotine delivery products.

Improving community understanding is not something that can occur in isolation from existing beliefs. We suspect that beliefs about the harmfulness of tobacco, and perhaps of negative assumptions about “chemicals” may have created a context where there is an initial assumption that anything labelled tobacco or which is a chemical (e.g. nicotine) is likely to be harmful. In such a context, it is clearly going to take more than reading one Fact Sheet, however persuasive, to convince many that their preconceptions are wrong. To proceed much further, we need a jurisdiction to try to systematically educate its smokers using reliable information, presented consistently through a concerted health education program. Sweden would be the ideal country to do this as it has a lot of ST users and use of NRT is also widespread. (More countries could readily host such a campaign limited to NRT). When such an educational campaign happens, it will be important to carefully evaluate its effects to see if it actually motivated increased movement away from smoked tobacco products.

In conclusion it is possible to provide smokers with information that improves their understanding of the relative harms of nicotine delivery products, but this does not necessarily translate into increased reported likelihood of use. The current lack of knowledge would seem to be largely due to the reluctance of governments to ensure that smokers are better informed about the mechanisms by which their dependence on nicotine is harming their health. This could be remedied either by requiring or encouraging manufacturers to publicise this information, and/or by governments doing so themselves through public information campaigns.

## Competing interests

The authors declare that they have no competing interests.

## Authors’ contributions

RB initiated the study which KM and AM developed for implementation in the UK, ROC and KMC adapted it for the US, LR and TW did so for Sweden, and BK and LL helped RB adapt for Australia and managed the implementation. LL and KM led the data analysis. RB developed an initial draft of the Fact Sheet. All authors contributed to the analysis and interpretation of the data and writing of the paper. RB is guarantor of the data. All authors read and approved the final manuscript.

## Supplementary Material

Additional file 1 **FACT SHEET.** Tobacco, Nicotine and health harm [1,][2,][4,][11,][30]. Click here for file
