# Supplementary material for: Effects of a Fact Sheet on beliefs about the harmfulness of alternative nicotine delivery systems compared with cigarettes
Source: Harm Reduct J. 2012 Jun 11;9:19. doi: 10.1186/1477-7517-9-19 (PMC3514329; doi:10.1186/1477-7517-9-19)
Supplement: Additional file 1 — FACT SHEET. Tobacco, Nicotine and health harm [1,][2,][4,][11,][30]. [file 1477-7517-9-19-S1.doc]

# Additional file 1. The Fact Sheet (Australia version)

# FACT SHEET: Tobacco, Nicotine and health harm

We all know that smoking tobacco is harmful. However, recent research has shown that some tobacco products are far less harmful than cigarettes. It has also shown that nicotine is not a major cause of long-term health harms, even though it is the drug in the tobacco that makes it addictive, and is the main reason people use tobacco.

The general expert consensus with smoked tobacco is that they are all very harmful and it is the smoke itself that is especially dangerous. They are somewhat less harmful if you don’t inhale the smoke into your lungs, but only take it into your mouth. Cigarette smokers need to take the smoke into their lungs in order to get enough nicotine to satisfy them. Pipe and cigar smoke is different to cigarette smoke and it is possible for pipe and cigar smokers to get a satisfying amount of nicotine through the mouth. Thus many pipe and cigar smokers do not inhale smoke into their lungs. However, cigarette smokers who switch to pipes or cigars generally continue to inhale the smoke into their lungs. Given this, there is little or no benefit in cigarette smokers switching to pipes or cigars.

The situation is different with smokeless tobacco products because they do not involve burning tobacco and smoke. Most of these are used by putting the tobacco into your mouth, so that the lungs are not affected. While most smokeless tobacco products are less harmful than smoked tobacco products, many traditional forms of smokeless tobacco (common in India, for example) cause mouth cancers and a range of other diseases and are still unacceptably harmful.

However, beginning in Sweden in the 1970s, some manufacturers began removing as much as they could of the harmful chemicals from their smokeless tobacco products. It is now possible to manufacture smokeless tobacco products with low levels of these chemicals. It is thus reasonable to expect that they might be less harmful. Recent research on large numbers of smokeless tobacco users shows that these newer smokeless tobacco products are indeed less harmful than older ones. The research shows that these cleaner forms of smokeless tobacco do not cause lung cancer and are unlikely to cause mouth cancer, but may still cause pancreatic cancer. They do not cause chronic bronchitis, because there is nothing taken into the lungs, which is what causes this disease. They may still cause some heart disease, but much less than smoking. Smokeless tobacco also causes some mouth ulcers and causes, or contributes to, some other health problems, so it is not a safe product to use long term. In particular, smokeless tobacco should not be used during pregnancy.

A recent expert panel (Levy et al, 2004) estimated that the risks of using these cleaner forms of smokeless tobacco were around one tenth of the risks of cigarette smoking. Other expert groups have suggested the risks of using smokeless tobacco are lower still. NB: The evidence does not support claims that smokeless tobacco use is safe. We would not encourage non-tobacco users to take it up. Indeed, we would recommend that they do not. However, if existing smokers were to switch to such products, the available evidence is that it would reduce their health risks a lot.

Nicotine, in the form of Nicotine Replacement Therapy (NRT), is a pure form of nicotine used to help people quit smoking. It is likely that NRT is even less harmful than smokeless tobacco, although there are health risks associated with any long-term nicotine use. NRT, however, may not be as attractive to smokers as an alternative to cigarettes, because current products are designed to minimize the pleasurable aspects of nicotine use, so people using them to quit will not be motivated to continue to use them longer term.

### Some commonly asked questions

**Why should we believe all this?**

There are good reasons why smokeless tobacco products could be less harmful than smoked tobacco products. There are three sources of the harmful chemicals in smoked tobacco. Most are produced as a result of the burning (combustion). Others are produced during the processes of curing and manufacture; and some are in the tobacco itself. Use of smokeless products only involves taking in the last two sources of harmful chemicals, and less chemicals may mean less harm.

Scientists have also found ways of reducing the levels of harmful chemicals in smokeless tobacco, but have not been able to do the same for tobacco smoke. Taken together, this means that smokeless tobacco starts off containing fewer harmful chemicals than cigarette smoke and there are practical ways for manufacturers to reduce them further.

Finally, our lungs are more sensitive than our mouths or stomach, so anything taken into the lungs is likely to cause more problems than similar things just taken into the mouth or stomach.

In the case of cigarettes, we have by far the most dangerous mix of harmful chemicals delivered to one of our most vulnerable organs. This all explains why the cleaner forms of smokeless tobacco are being found to be a lot less harmful.

**Is nicotine really OK?**

Nicotine is a poison and can kill you if you take enough of it. However, when taken in the doses that people knowingly take in, it is metabolized rapidly enough so it does not build up to dangerous levels in your body. Tobacco users rarely overdose, as there is a large gap between a dose that is experienced as enjoyable and a dose that is dangerous. In between, there is a level experienced as unpleasant and/or nauseating, which almost always leads users to stop before they really poison themselves.

We simply do not know how harmful long-term use of pure nicotine is. Not enough people have used pure forms of nicotine for long enough to know. Our guess, and that of most experts is that it is probably less harmful then even the cleanest forms of smokeless tobacco. For somebody who does not use tobacco, there are risks associated with using any form of nicotine, health risks as well as the risk of becoming addicted. However, for a smoker, who currently has all of those risks, plus many more, it could be a means of getting rid of many of the other risks, making them much better off.

Companies that market nicotine replacement products to help smokers quit have spent some effort trying to explain that nicotine is not the main cause of the disease smoking causes, but most people still have not got that important message.

**But isn’t addiction a bad thing?**

Yes, nobody would argue that being addicted to something is good. But addiction does not cause ill-health by itself. It contributes to the ill health caused by smoking because it is what keeps smokers continuing to take the harmful chemicals into their bodies for the long periods it often requires for diseases to develop. If there are no harmful chemicals, then these kinds of harms would not occur. The main direct harm from addiction is economic. An addiction causes you to spend money that might be better spent in other ways. For less well off smokers this can mean money spent on tobacco that would have been better spent on good food and other things that maintain health. Thus for the poor, in particular, addictions can be an indirect cause of ill-health. On the other hand, some people claim to get benefits from using nicotine. While addiction might never be good, there may be circumstances when using an addictive product is acceptable. Ultimately this is a value judgment, and a risk that some may consider worth taking.

**Why isn’t the information that smokeless is less harmful being given to smokers?**

The information has not been withheld from smokers. However, because these products are not available in Australia, the information has probably not been seen to be newsworthy, and thus isn’t mentioned much. There are a number of other reasons for it not being well known, some related to the knowledge being relatively new. Until recently, researchers looking at the health effects of smokeless tobacco products had not focused on differences between products. The smokeless tobacco products around when earlier research was conducted clearly caused cancer and other problems. Thus, it was widely agreed that smokeless tobacco was harmful and should be discouraged. Because smoking dominates tobacco use around the world, most of the attention has been on smoking. The efforts to make cigarettes less harmful, through such things as adding filters and trying to reduce tar levels have failed. This made people wary of the idea of less harmful tobacco products.

Further, making comparisons of harmfulness are only really relevant if the less harmful product is seen as a real alternative to the more harmful one. There has been a widespread belief that smokers will generally not be interested in switching to smokeless products or able to do so. In Sweden, there has been a considerable amount of switching to smokeless tobacco over the years, particularly among men. Reports of this have led some to ask whether this is likely to happen elsewhere.

If smokers were not interested in using smokeless tobacco but a proportion of non-smokers took it up, then promoting smokeless tobacco could result in an increase in harm at the population level. This has been a major concern. Very recently, research from Sweden has been published that indicates smokeless tobacco is not associated with uptake of cigarettes and that it may play a role in helping smokers to quit. In Sweden many smokers use smokeless tobacco as a step to quitting tobacco use altogether, while others transfer to just using smokeless tobacco. More Swedish men use smokeless tobacco than would probably use smoked tobacco products in a “smoker-only” market (such as currently exists in Australia).

Finally, there are real concerns that tobacco companies would exploit public statements that smokeless is far less harmful to imply that it is entirely harmless or that the risk is so slight as to be not worth worrying about. If this happened, it could result in a large increase in use of smokeless tobacco and an increase in harm, unless it resulted in less smoking.

**References:**

1. Foulds J, Ramstrom L, Burke M, Fagerstrom K (2003). Effect of smokeless tobacco (Snus) on smoking and public health in Sweden. *Tobacco Control* 12:349-359

2. Rodu & Jansson, (2004) Smokeless tobacco and oral cancer: A review of the risks and determinants. *Crit Rev Oral Biol Med*. 15:252-263

4. Levy et al, (2004) The relative risks of a low-nitrosamine smokeless tobacco product compared with smoking cigarettes: Estimates of a panel of experts. *Cancer Epi Biomarkers Prev*. 13:2035-2042

11. Ramstrom L, Foulds J (2006). Role of snus in initiation and cessation of tobacco smoking in Sweden. *Tobacco Control* 15:210-214.

30. Broadstock M (2007) Systematic review of the health effects of modified smokeless tobacco products. New Zealand Health Technology Assessement, Christchurch. Vol 10 No. 1.
